# Peer Rejection and Group Autonomy in the Latency Stage: A Qualitative Analysis of Children’s Voices in the Classroom Context

**DOI:** 10.3390/children13040463

**Published:** 2026-03-27

**Authors:** Hana Fisher-Grafy, Yael Malin

**Affiliations:** 1 Emotional-Social Psychotherapy Program for Children, Bar Ilan University, Ramat-Gan 5290002, Israel; hamania10@gmail.com; 2 Max Planck Institute for Human Development, 14195 Berlin, Germany

**Keywords:** social rejection, latency stage, peer group, group autonomy, social status, collective identity, qualitative research

## Abstract

**Highlights:**

**What are the main findings?**

- Children in the latency stage demonstrate a shared developmental drive for classroom-based group autonomy and collective decision-making.
- Group autonomy functions as an implicit hierarchical criterion shaping social status, with higher status associated with alignment to peer norms and reduced reliance on adults.

**What are the implications of the main findings?**

- During the latency stage, autonomy shifts from an individual capacity to a collectively organized peer-group process.
- Understanding group autonomy as a developmental dimension provides a new framework for explaining social status and peer rejection, and guides the development of practical classroom-based educational interventions.

**Abstract:**

**Background/Objectives:** Social rejection during the latency stage is a significant risk factor for children’s emotional and social development. Whereas earlier research focused primarily on individual characteristics of rejected children, contemporary perspectives emphasize peer-group processes, including norm formation, hierarchies, and social status structures. Although autonomy has been widely examined as an individual developmental construct, less attention has been given to its possible collective expression within the classroom peer group. This study aimed to explore how children understand and experience group autonomy and to clarify its role in social status and peer rejection. **Methods:** Twelve classroom-based focus groups were conducted with 140 fifth-grade children from five public elementary schools in Israel. Discussions were initiated using a projective narrative describing ambiguous peer exclusion. Data were audio-recorded, transcribed verbatim, and analyzed using thematic analysis. Coding was conducted independently by two researchers and refined through iterative comparison and reflexive procedures. **Results:** Three themes emerged: (1) a shared longing for classroom-based group autonomy and collective decision-making; (2) group autonomy as an implicit hierarchical criterion shaping social status, whereby reduced reliance on adults and alignment with peer norms were associated with higher status, while adult dependence was linked to marginalization; and (3) an ambivalent structure of autonomy, as children valued peer independence yet expressed fear of adult punishment and responsibility. **Conclusions:** Findings suggest that during the latency stage autonomy shifts toward a collectively organized peer-group process. Recognizing group autonomy as a developmental dimension may deepen understanding of social status and peer rejection within classroom contexts.

## 1. Introduction

### 1.1. Peer Rejection in Latency Age

Peer rejection is defined as a negative and enduring social status within the peer group, manifested in patterns of non-selection, avoidance, and social exclusion [1,2]. Comprehensive reviews and meta-analyses indicate a high prevalence of experiences of social victimization during childhood. Despite variability across studies due to differences in definitions, measurement methods, and sources of report, the overall picture is consistent: a substantial proportion of children experience rejection, bullying, or social exclusion during the elementary school years [3,4].

Moreover, a broad body of research points to the psychological implications of rejection and peer victimization including depression, anxiety, loneliness, decreased self-esteem, and impaired sense of belonging [4,5]. Longitudinal studies indicate that rejection is not merely a consequence of early emotional difficulties, but also a risk factor for their development and exacerbation over time, with bidirectional relations between emotional vulnerability and social vulnerability [6].

The consequences of peer rejection may extend beyond childhood. Population-based longitudinal studies indicate an association between experiences of rejection and bullying in childhood and increased risk for psychiatric disorders, impairments in social and emotional functioning, and reduced psychological well-being in young adulthood, even after controlling for confounding variables such as family difficulties or early psychopathology [7]. These findings reinforce the understanding that peer rejection constitutes a significant developmental risk factor.

Neuroimaging studies have shown that experiences of social exclusion activate brain regions also associated with the processing of physical pain, such as the anterior cingulate cortex and the insula [8]. These findings support the conceptualization of rejection as a genuine experience of “social pain” and underscore the centrality of social belonging to development and psychological well-being.

### 1.2. Autonomy as a Developmental Axis: From Infancy to the Latency Stage

Developmental research conceptualizes autonomy as a cumulative process of agency, initiative, and self-directed action that unfolds within relational contexts across childhood. Empirical findings consistently show that autonomy does not develop in opposition to connection but rather emerges from secure and autonomy-supportive relationships that enable exploration and independent action [9,10].

In infancy, autonomy is expressed primarily through exploratory behavior organized around the availability of a secure base. Classic attachment research demonstrated that securely attached infants engage in more confident and sustained exploration in the presence of a responsive caregiver action [11,12]. Longitudinal and meta-analytic findings further indicate that early attachment security predicts later social competence and adaptive independent functioning [9,13]. At this stage, autonomy is manifested as expanding initiative within a framework of secure relational dependence.

During toddlerhood, autonomy becomes behaviorally explicit as children increasingly assert choice, intention, and self-initiated action. Research on autonomy-supportive parenting shows that when caregivers encourage choice and acknowledge the child’s perspective, children display higher levels of independent functioning and internalized motivation [10,14]. Empirical longitudinal studies further demonstrate that early autonomy-supportive interactions predict later independent goal-directed behavior [15].

During the preschool years, autonomy expands beyond the capacity to “do alone” and becomes increasingly expressed through initiative, planning, and the active organization of play and social roles. According to Erikson’s [16], this period is marked by the tension between initiative and guilt, as children increasingly initiate activities, negotiate roles, and take leadership within pretend play and collaborative games. Empirical research on preschool peer culture supports this shift by showing that children’s play is organized through emerging participation rules, inclusion–exclusion practices, and negotiated access to group activities—processes that reflect early forms of socially situated agency guilt [17]. Complementing this, a systematic synthesis of research on leadership in preschool play highlights that leadership and initiative are observable and developmentally meaningful features of children’s peer play organization guilt [18].

With entry into formal schooling, autonomy is increasingly expressed through the ability to take responsibility for tasks, sustain engagement, and develop competence within institutional expectations. Research grounded in self-determination theory shows that autonomy-supportive practices by parents and teachers are reliably associated with higher intrinsic motivation, classroom engagement, and more adaptive academic and social functioning [19,20]. A large-scale meta-analysis further demonstrated that parental autonomy support significantly predicts academic achievement and a broad range of positive school-related outcomes, with particularly robust relevance during school transitions (Vasquez et al., 2016) [20]. In addition, experimental and intervention-based evidence indicates that when educators are trained to become more autonomy-supportive, students show improved motivational and engagement-related outcomes, underscoring the causal role of autonomy-supportive contexts in school adjustment [21].

### 1.3. The Conceptual Gap in Understanding Autonomy During Latency Age

Developmental literature describes autonomy as a gradual process that unfolds from infancy through adolescence. Early research focused on the establishment of personal competence and efficacy at the beginning of formal schooling [16], whereas later studies emphasized autonomy in decision-making and positioning vis-à-vis parental authority during adolescence [22,23]. Longitudinal research indicates a gradual increase in autonomy across middle childhood and adolescence, particularly in aspects of personal decision-making [24].

However, despite these contributions, the middle elementary school years—particularly grades 3–5 within the latency stage, have not received distinct theoretical conceptualization with regard to the development of autonomy. In most studies, this period is discussed as a direct continuation of early schooling, with an emphasis on functioning, persistence, and competence, yet without a qualitative distinction between the child’s personal autonomy and the collective processes unfolding within the peer group. In other words, the literature largely portrays a linear development of individual autonomy, while giving limited attention to the question of how the focus of autonomy may shift during a period in which the peer group becomes the primary developmental arena.

This gap is particularly significant in light of research emphasizing that during latency stage, the peer group serves as a central agent of socialization, and that group norms and social status become crucial for emotional and social adjustment [2,25]. If the peer group becomes an organizing framework for identity and belonging, the question arises whether autonomy itself changes its nature during this developmental phase. This consideration leads us to the proposal of a new developmental framing, suggesting that during the latency stage autonomy does not develop solely as an individual capacity of the child, but also as a form of collective autonomy of the children’s classroom community.

### 1.4. The Development of Group Autonomy in Latency Age: A Collective Perspective

Research consistently identifies the latency stage as a developmental period in which the peer group becomes a central arena for social organization, status differentiation, and norm formation [2,25]. During this stage, children increasingly orient themselves toward the classroom peer community as a meaningful social reference system. While autonomy has been extensively examined as an individual developmental construct, far less attention has been devoted to its potential collective manifestations within the peer group during middle childhood. Most of the research conceptualizes autonomy as self-directed functioning in relation to adults, focusing on parental and teacher autonomy support and its links to motivation, achievement, and adjustment [20].

The “From Me to We” framework [26,27,28,29] seeks to extend this perspective by conceptualizing autonomy during latency age as a developmental group process in which the classroom peer group increasingly functions as a socially organizing unit with its own internal norms, shared identity, and capacity for collective decision-making. Within this process, authority gradually shifts in relative terms from adult-centered regulation toward internally negotiated peer regulation. This shift does not imply the disappearance of adult influence; rather, it reflects a reorganization of the child’s social world in which the peer group becomes a significant source of social authority.

Within this conceptualization, group autonomy represents a qualitative shift beyond individual independence. It refers to the emergence of collective agency, where the group itself becomes an organizing structure that shapes expectations, regulates behavior, and defines participation. Social status, in this view, reflects the child’s degree of integration into the group’s collective movement toward shared norms and coordinated functioning. Children who successfully align with and contribute to this collective organization tend to consolidate their social position, whereas those who remain positioned primarily in relation to adult authority or outside the group’s internal coordination may be at increased risk for marginalization or rejection.

Preliminary quantitative examination of components of this framework [28] identified significant associations between social status and adaptation to group norms, as well as between social status and behavioral alignment with group flow. However, aspiration for autonomous group decision-making did not emerge as a statistically distinct correlate of social status. This discrepancy between the theoretical centrality of group autonomy and its limited quantitative differentiation highlights the need for deeper conceptual and empirical clarification. The relative absence of empirical attention to autonomy as a group-level developmental phenomenon suggests a significant gap in the literature.

## 2. Aim of the Study

The present study seeks to examine how children in the latency stage articulate, understand, and experience group autonomy within the classroom context, and to clarify its role in the development of social status and peer rejection.

### Research Questions

1. How do children in latency age experience and make sense of social acceptance and rejection within their classroom?
2. What explanations and meanings do children attribute to differences in social status among classmates?
3. How do children describe and interpret their classroom’s sense of independence as a peer group within the school context?

## 3. Method

### 3.1. Research Design

The present study was conducted using a qualitative approach, employing focus group methodology in order to examine dynamic social processes as they unfold within the natural classroom environment of children in latency age. This method was found to be particularly suitable for research aiming to explore children’s subjective experiences, perceptions, and interpretations, as well as real-time interactions and group dynamics, in the context of reduced dependence on adults, autonomous social decision-making, and peer rejection. Focus groups enabled the researcher to integrate into the flow of group discourse and to conduct close observation of natural social processes occurring within the peer group [30,31,32].

### 3.2. Participants

Participants were 140 children in Grade 5 (aged 10–11 years) who took part in 12 focus groups. Participants were recruited using a snowball sampling

Recruitment was initiated through educational–psychological services, school counselors, and school principals who facilitated access to schools. This approach enabled the identification of classrooms representing diverse social contexts while maintaining ecological validity.

The focus groups were conducted in five public elementary schools in Israel representing diverse social contexts. Six groups were drawn from schools located in central towns and cities and six from rural communities. In addition, three groups were drawn from state religious schools and nine from state secular schools. Participants came from schools serving children from typical middle socioeconomic backgrounds.

Gender composition varied across groups. Three focus groups consisted only of girls, one consisted only of boys, and the remaining eight groups were mixed-gender. Among the mixed groups, one included approximately 25% boys and 75% girls, another included approximately 80% boys and 20% girls, and the remaining groups included roughly equal numbers of boys and girls.

Participation was voluntary and included only children whose parents provided written informed consent. Consequently, focus groups included only a portion of each classroom, as children whose parents did not provide consent did not participate.

Inclusion criteria consisted of children enrolled in regular fifth-grade classrooms who were able to participate in group discussions. Exclusion criteria included children who did not receive parental consent or who were not present on the day of data collection. No additional exclusion criteria were applied in order to preserve the natural composition of the classroom social context.

### 3.3. Composition of the Focus Groups

A total of 12 focus groups were conducted: three groups from state religious schools and nine from state secular schools. In terms of gender, eight groups were mixed-gender groups, two were boys-only groups, and two were girls-only groups. The groups included between 15 and 22 participants. The relatively large size of several focus groups reflected the theoretical premise of the study. The research was based on the assumption that during the latency stage the classroom functions as the primary social arena in which a collective social self develops within the peer group. For this reason, it was important to observe the social discourse within the natural peer context of the class rather than within artificially reduced groups. Although larger focus groups may limit individual speaking time, in practice the children tended to express themselves in short statements and spontaneous exchanges, allowing multiple voices to emerge during the discussion. Conducting focus groups that approximated the classroom structure therefore enabled the researchers to capture the dynamic interplay between individual perspectives and collective peer norms.

### 3.4. Procedure

All focus groups consisted of participants from the same classroom. This structure was based on findings in the literature indicating that focus groups are particularly effective when participants share a common social identity and shared experiences [31]. This design enabled discussions grounded in authentic group dynamics and shared social language. The groups were conducted within the familiar school environment in order to reduce situational anxiety and to facilitate relatively natural discourse. All sessions were audio-recorded and fully transcribed.

Each discussion began with a short projective narrative describing a classroom that did not invite two children to a birthday party. The story was intentionally formulated in a relatively ambiguous manner, without specifying an explicit reason for the exclusion, in order to allow children to project their personal and collective interpretations onto the scenario. The use of a narrative-projective stimulus in qualitative research with children has been found to be particularly effective in examining sensitive social topics, as it enables indirect expression of attitudes and group norms while reducing social desirability bias and defensive mechanisms [33,34]. The story served as a trigger for open discussion and did not include any explicit reference to parents or teachers.

### 3.5. Data Analysis

All focus group discussions were audio-recorded and transcribed verbatim. The transcripts comprised approximately 98 pages of text documenting about 13 h of recorded discussions.

The data were analyzed using the modified Van Kaam method described by Moustakas (1994) [35], a phenomenological analytic approach designed to identify essential meanings within participants’ experiences. The analytic process followed several systematic stages. First, both researchers independently read the transcripts repeatedly in order to become familiar with the data. Second, all statements referring to peer exclusion were identified. Third, statements that were not directly related to peer exclusion were eliminated. Fourth, the remaining statements were grouped into preliminary themes reflecting recurring patterns in the participants’ explanations. Fifth, each researcher constructed descriptive accounts of the emerging themes across the focus groups. Sixth, the themes were organized into broader categories reflecting both individual-level and group-level processes. Finally, the researchers jointly developed a structural description of the phenomenon, identifying three central themes that captured the children’s interpretations of peer exclusion.

To enhance the credibility of the analysis, both researchers conducted the coding process independently and subsequently compared their interpretations. Differences in coding were discussed until agreement was reached. Reflexive procedures were also implemented to increase analytic transparency. Prior to the analysis, each researcher explicitly identified their assumptions regarding peer exclusion. The first researcher, an educational psychologist working with socially rejected children, approached the phenomenon from a developmental perspective, whereas the second researcher, an educational counselor working in schools, viewed peer exclusion primarily as a class-level social dynamic. Articulating these assumptions allowed the researchers to remain aware of potential interpretive biases throughout the analytic process. The use of independent coding, comparison of interpretations, and reflexive awareness contributed to the trustworthiness of the findings. These procedures were intended to enhance the rigor and trustworthiness of the analysis. Independent coding by two researchers, comparison of interpretations, reflexive documentation of analytic decisions, and internal peer debriefing were used to strengthen the credibility and transparency of the findings.

In addition, reflexive memos were documented throughout the analytic process in order to systematically track interpretive decisions and to enhance transparency regarding the researchers’ influence on data interpretation.

### 3.6. Ethical Considerations

The study received approval from the relevant institutional ethics committee (No. 10284). Informed consent was obtained from parents, and active assent was obtained from the children themselves. Given the potential sensitivity of the topic of peer rejection, measures were taken to prevent the identification of specific children during discussions. The researcher intervened in cases where concern arose regarding potential harm to a particular child or direct accusation.

## 4. Findings

The analysis of the focus group data revealed three central themes reflecting a complex developmental structure of group autonomy in latency age:

1. A need and longing for group autonomy
2. Group autonomy as a hierarchical criterion determining social status
3. The dual and ambivalent structure of autonomy, limiting its full realization

The following sections elaborate each of these themes in detail.

### 4.1. Theme 1: Need and Longing for Group Autonomy

The first theme reflects a clear need among children for group autonomy and decision-making within the classroom peer society. The children describe themselves as “old enough,” emphasize their belonging to an older grade, and express a desire to act without constant adult supervision.

Emily described the group’s sense of maturity and the legitimacy of independent action, even at the cost of punishment:

“Because he thinks he’s independent enough to decide things on his own and doesn’t need parents watching him all the time… like, they’re in fifth grade, they’re big already, and they can make decisions themselves. They’ll say, ‘I did the right thing.’ So fine, I’ll take the punishment.”

Notably, although the child initially refers to an individual classmate (“he”), the explanation quickly shifts to a collective frame (“they’re in fifth grade… they can make decisions themselves”). This movement reflects a developmental transition from individual autonomy to classroom-based collective autonomy, experienced as interconnected rather than separate. The speaker does not distinguish sharply between the independence of a single child and that of the class as a whole; instead, individual self-determination appears embedded within a broader shared identity. Such formulations suggest that, during latency age, personal autonomy and group autonomy are perceived as mutually reinforcing dimensions of the same developmental process.

Liam articulated the class’s right to decide as self-evident:

“In the class there are things they decide, so I think that’s something they should be able to decide, and they should listen and do what they want.”

When asked why it was important for children to decide for themselves, responses were simple and direct:

“Because it gives them independence.”

“Because they’re the ones deciding.”

Autonomy appears here as a value in itself.

In the context of a unanimous class decision, Ava explained:

“Because the class decided together and they said they didn’t want to… and that’s what they decided, so they said, ‘That’s what we decided.’”

The emphasis was not on the content of the decision, but on the fact that it emerged from collective agreement.

An emotional peak of this theme appeared when Sophia expressed a longing for a strong and united class:

“A really strong group, and that we can do everything together, and not always, ‘Oh, I don’t want to be with him, who is he?’…”

These findings indicate that the aspiration for group autonomy constitutes a shared collective developmental need.

### 4.2. Theme 2: Group Autonomy as a Hierarchical Criterion—Social Status as Degree of Participation

The second theme clarifies that group autonomy is not merely a general aspiration but a clear hierarchical criterion determining social status. Status within the class is shaped by the child’s degree of participation in the group’s movement toward autonomy, particularly through reduced dependence on adults.

#### 4.2.1. Dependence on Adults as a Violation of the Group Code

The most critical and emotionally charged responses were directed toward children who involved adults in peer matters. In the children’s accounts, turning to parents or teachers was not described merely as a behavioral choice, but as a breach of an implicit group code.

Ethan stated:

“I think it’s because they’re snitches… about every little thing they go tell.”

Noah explained:

“If they listen to their parents, people will think they’re less ‘popular,’ and then… they won’t invite them to parties.”

These statements suggest that reliance on parental authority is interpreted within the peer culture as a sign of reduced social standing. In the children’s narratives, popularity is implicitly tied to the capacity to act independently of adult oversight. Dependence on adults is therefore not neutral; it signals misalignment with the group’s shared orientation toward autonomy and may lead to exclusion, symbolically expressed through not being invited to social gatherings and more broadly through marginalization within the peer group.

Mason added:

“If he thinks about the class and not about his parents, he becomes more popular. If he thinks about his parents, he thinks about all the rules…”

Mason’s formulation makes this hierarchy explicit. Social status is presented as contingent upon the child’s orientation toward the peer group rather than toward adult authority. “Thinking about the class” signifies alignment with the collective norms and priorities of the group, whereas “thinking about parents” signifies adherence to externally imposed rules. Within this interpretive framework, popularity is granted to those who demonstrate loyalty to the children’s shared code and who privilege group-based judgment over adult regulation. The more a child is perceived as guided by the group’s internal logic, the more they are recognized as belonging and, consequently, as socially valued within the classroom community.

This valuation is further reinforced in children’s spontaneous reactions to acts of defiance toward parental authority. As one child expressed:

“Wow, that’s cool—you didn’t listen to your parents.”

Such responses frame resistance to adult control not merely as acceptable, but as admirable. Disregarding parental directives is constructed as a sign of courage and maturity, eliciting admiration from peers. Within this social logic, distancing oneself from adult authority becomes a symbolic marker of alignment with the group’s autonomous identity and a pathway to enhanced social recognition.

At the same time, children who insist on their own preferences and fail to align with the group’s direction are described in markedly negative and infantilizing terms. One girl explained:

“They behave kind of like babies. If they’re told to play a certain game, they go ‘Ugh!’ and start crying and walking away like they’re three years old. Or if we do something they want, they start jumping and screaming ‘Yes! Yes! Yes!’”

#### 4.2.2. Maturity Versus “Childishness” in Social Context

In discussions of games and competition, a distinction emerged between children who regulate themselves independently and those who turn to adults.

Mason observed:

“They (the high-status kids) win with respect and lose with respect…”

Lucas added:

“I’d shake hands and say, ‘Good game.’”

Daniel elaborated:

“Most of the time, when you lose with respect, you become more popular, not if you cry…”

Crying, taking things too personally, or turning to adults were framed as signs of “childishness.”

Thus, group autonomy, especially reduced dependence on adults, functions as a central criterion in the classroom’s hierarchical structure.

Taken together, the children’s accounts suggest a recognizable pattern in the way social standing is described within the classroom. Popularity appears to be associated with thinking about the class rather than about parents (“If he thinks about the class and not about his parents, he becomes more popular”), going along with the group’s decisions, and avoiding turning to adults in peer matters (“about every little thing they go tell”). Children who are perceived as able to accept group choices and manage disappointment without overt protest are often spoken about in more approving terms, sometimes even with admiration when they distance themselves from parental authority (“Wow, that’s cool—you didn’t listen to your parents”).

By contrast, those who involve adults, insist strongly on their own wishes, or respond with visible crying are described in more infantilizing language, such as “like they’re three years old,” and are portrayed as less popular (“they won’t invite them to parties”). While these patterns are not presented by the children as explicit rules, their narratives suggest that social standing may be shaped, at least in part, by how closely a child is seen to align with the group’s shared expectations and ways of acting.

### 4.3. Theme 3: The Ambivalent Structure of Group Autonomy

Alongside the clear valuation of autonomy, an ambivalent structure emerged. When autonomy involves responsibility and real consequences, anxiety arises.

Harper explained:

“Kids should be independent… but it’s not always good if they decide, because they might decide the wrong things.”

Mia reflected:

“I think they would tell their parents the truth and then get punished…”

Autonomy is recognized as a value, yet it is associated with risk.

At the same time, adult messages themselves destabilize children’s confidence in expressing autonomy.

Grace stated:

“Because they educate us that they’re (the parents) always right.”

And Chloe reflected:

“Because their parents have always told them since they were little to listen to them…”

Alexander said:

“Good kids listen to their parents… whatever parents say, you have to listen.”

A dissonance becomes evident: the group values autonomy and reduced dependence on adults, yet children fear making mistakes, fear punishment, and fear violating parental expectations of obedience.

This tension reveals the dual structure of group autonomy:

On the one hand, reduced dependence on adults is socially rewarded and strengthens status. Children who frequently involve adults are positioned lower in the hierarchy (often labeled “snitches”), whereas those perceived as acting independently are admired and even glorified (“brave”).

On the other hand, autonomous decision-making involving responsibility generates hesitation and fear, particularly fear of adult reaction and fear of undermining parental education that emphasizes obedience.

### 4.4. Summary of Findings

Taken together, the three themes suggest that group autonomy constitutes a salient and developmentally meaningful orientation during the latency stage. The findings indicate that the classroom peer group is characterized by a shared aspiration toward functioning as an autonomous collective, a striving that appears widespread and normative at this developmental stage. Within this process, social status emerges as structured around children’s perceived contribution to the consolidation and advancement of collective autonomy. At the same time, the expression of autonomy is marked by ambivalence: although autonomy is explicitly valued and socially rewarded, particularly as a marker of maturity and peer alignment, it is simultaneously experienced as risky when it entails responsibility and real consequences. Children articulate anxiety about making mistakes, being punished, and violating internalized expectations of obedience, revealing that the pursuit of collective autonomy is accompanied by tension and hesitation. This ambivalence may constrain the full behavioral realization of group autonomy despite its strong developmental presence.

## 5. Discussion

The present qualitative study examined how children in the latency stage articulate and experience Group Autonomy within the classroom context, and how these experiences are integrated into their perceptions of social status and peer rejection. The findings indicate that classroom-based Group Autonomy constitutes a significant developmental component at this stage. Children express a clear longing for the class to function as an autonomous unit, to make decisions together, and to reduce dependence on adults. At the same time, the actual expression of this autonomy is restrained due to fear of adult reactions, punishment, and the violation of parental expectations of obedience.

### 5.1. Classroom Based Group Autonomy as a Developmental Aspiration in the Latency Stage

The first theme indicates that children perceive latency age as a stage in which the class is capable of functioning independently. Repeated references to being ‘like adults,’ making decisions independently, and wanting to be a ‘strong class’ reflect a developmental shift toward establishing the classroom as a legitimate children’s space with its own internal authority.

This autonomy is not described merely as individual independence but as classroom-based Group Autonomy, namely the capacity of the class to decide, agree, and act according to internal norms rather than solely external authority. This finding is consistent with Piaget’s description of the transition from heteronomous morality grounded in external authority to autonomous morality based on reciprocity and norms constructed within peer interaction [36]. Later empirical research on moral development during the school years likewise demonstrates that children increasingly attribute legitimacy to group agreements and peer-based reasoning within the social domain [37,38]. Accordingly, classroom-based Group Autonomy may be understood as a normative developmental process in which the peer group becomes a meaningful source of social authority.

### 5.2. Group Autonomy as a Hierarchical Criterion of Social Status

The second theme clarifies that Group Autonomy is not merely a general aspiration but a hierarchical principle organizing social status within the classroom. The children’s accounts indicate clearly that the more a child expresses independence in relation to adults, refrains from turning to parents or teachers, acts according to the group code, and prioritizes the class over personal wishes, the more that child is perceived as mature and socially valued. Children who act without adult intervention are described as brave and even as heroes. Acts of resistance to parental authority in social situations are met with admiration and excitement from peers. In contrast, children who turn to adults in order to impose their personal wishes, who report to teachers or parents, or who react with crying and visible dependence are labeled childish or snitches and are described as declining in social standing, sometimes to the point of social rejection.

The principle emerging from the findings is clear. Social status is closely tied to the degree to which a child embodies and promotes the Group Autonomy of the classroom. The more a child aligns with the group’s independent functioning and reduces reliance on adult authority, the higher the social recognition. Conversely, the more a child relies on adults to regulate peer situations or enforce personal desires, the lower the perceived maturity and status. In this sense, Group Autonomy functions as an implicit measure of social maturity within the classroom hierarchy. Although gender differences were not the primary focus of the present study, the variation in focus group composition suggests that the expression of Group Autonomy and the criteria for social status may differ between boys and girls. It is possible that boys’ groups may place greater emphasis on visible independence and assertive behavior, whereas girls’ groups may rely more on relational coordination and social cohesion. Future research should further examine whether gender moderates the ways in which Group Autonomy is constructed and translated into social status.

### 5.3. Fear of Expressing Group Autonomy

The third theme highlights a significant tension. Alongside the clear longing for Group Autonomy, children express a concrete fear of fully expressing it. This fear stems from anticipated punishment, concern about making incorrect decisions, and the internalized message that parents are always right and must be obeyed.

Children are aware that classroom-based Group Autonomy may conflict with parental education and expectations. Thus, even though Group Autonomy is valued within the peer group, its expression is constrained by continued loyalty to parental authority. The longing for Group Autonomy is present and meaningful, yet children hesitate to express it openly and consistently due to fear of consequences. These dynamics may be particularly challenging for children with developmental difficulties, such as attention regulation, social communication, or emotional regulation challenges. Such children may find it more difficult to interpret or align with the implicit “group code” of autonomy, which may increase their risk of marginalization within the peer group. This perspective highlights the importance of educational mediation that bridges individual differences and group expectations, enabling broader social participation.

### 5.4. Reconciling the Gap with the Quantitative Findings

The qualitative findings provide a refined understanding of the gap identified in the previous quantitative study [28]. If children experience a genuine longing for Group Autonomy yet simultaneously fear expressing it because of potential adult reactions, then direct quantitative measurement of aspiration for autonomy may fail to capture its full developmental significance.

In other words, the absence of a significant quantitative association does not necessarily indicate that Group Autonomy is not a major factor related to social status and rejection. Rather, it may reflect restraint in its overt expression. Children may avoid consistently declaring or endorsing autonomous positions, despite the fact that Group Autonomy functions as a meaningful organizing principle of classroom social life.

### 5.5. The Theoretical Contribution of the Study

The findings propose a meaningful expansion of the understanding of autonomy development during the latency stage. While developmental literature has addressed the continuity of autonomy from infancy through early childhood and into adolescence, the primary emphasis has been placed on the autonomy of the individual child in relation to authority figures. The present study suggests that during the latency stage, an additional and qualitatively distinct developmental process takes place—the development of Group Autonomy.

This autonomy is not a quantitative extension of individual independence but a shift in the developmental center of organization. The child does not merely learn to function without reliance on adults; rather, the child learns to function as part of a group that aspires to its own autonomy. The locus of autonomy moves from the individual to the peer group, and the group becomes an entity with its own will, norms, and capacity for internal decision making. In this sense, the latency stage is not solely a stage of consolidating personal competence but a stage in which children develop the capacity to participate in the advancement of the group’s autonomy. Autonomy becomes organized as a shared space—the child contributes to the development of the class’s autonomy, and the class in turn provides a framework of belonging and social positioning.

The theoretical significance of this finding is twofold. First, autonomy continues to develop during the latency stage, but it changes in form. Second, it is necessary to distinguish between individual autonomy and Group Autonomy, as they operate according to different social logics. Group Autonomy is not limited to reduced dependence on adults; it involves the capacity of the peer group to develop internal norms, assume shared responsibility, and function as an autonomous unit within the school framework. Placing the group rather than the individual child at the center shifts the understanding of processes such as social status and rejection. Status is not solely a function of personal traits, but of the child’s degree of participation in and contribution to the development of the group’s autonomy. In this sense, Group Autonomy may also be understood as involving higher-order thinking skills (HOTS), as it requires children to engage in processes of evaluation, negotiation, perspective-taking, and collective decision-making. Thus, group functioning reflects not only social development but also the emergence of complex cognitive processes within peer interactions. In contemporary contexts, peer group dynamics increasingly extend into digital environments, where communication, visibility, and social influence are shaped by technological platforms. These environments may further amplify or transform the ways in which Group Autonomy is constructed, negotiated, and regulated within peer groups. Future research may examine how digital communication and constructs such as technological self-efficacy influence collective agency, internal group norms, and the regulation of social status.

### 5.6. Educational Implications

If Group Autonomy constitutes a central developmental process during the latency stage, then the educational task is not limited to fostering individual independence, but also to enabling the classroom group to develop as an autonomous unit. This does not imply relinquishing adult authority. Rather, it calls for a recalibration of its function.

However, supporting Group Autonomy presents a complex challenge for educators. Teachers are required to balance the encouragement of independent group processes with the need to maintain institutional norms, classroom management, and safety. This tension may create uncertainty regarding the appropriate level of adult intervention and highlights the need for professional tools to navigate these competing demands.

Educators are not required to suppress or negate the group’s aspiration for autonomy through excessive control, threat, or an exclusive emphasis on obedience. Instead, they are invited to create a secure space in which the group can practice decision making, construct internal norms, and gradually assume responsibility without fear of immediate punishment for every mistake.

Supporting Group Autonomy includes:

- Providing genuine opportunities for classroom decision making rather than merely symbolic participation
- Recognizing the legitimacy of internal group norms as long as they do not cause harm
- Reducing immediate adult intervention in every peer conflict
- Framing responsibility as a developmental process rather than a disciplinary issue

When adults support this process, Group Autonomy does not develop in opposition to authority but through a contained and gradual maturation. Conversely, when educational messages emphasize obedience and fear of error in rigid terms, the group may refrain from expressing its autonomy even when the developmental longing for it is present. Accordingly, educators are encouraged to regard Group Autonomy as a developmental resource to be consciously cultivated rather than as a deviation to be restrained. Careful, consistent, and trust-based support may allow the classroom group to mature in its autonomy without generating unnecessary anxiety or confrontation with the adult world.

### 5.7. Limitations

This study has several limitations that should be acknowledged. First, the use of a qualitative design and purposive sampling limits the generalizability of the findings. Second, the focus group format, particularly with relatively large groups, may have constrained the depth of individual expression and may have been influenced by dominant voices within the group. Third, the study was conducted within a specific cultural and educational context (Israeli elementary schools), which may limit the transferability of the findings to other cultural settings. Finally, the reliance on self-reported group discourse may reflect normative representations rather than actual behavior. Future research may benefit from combining qualitative and quantitative approaches and from examining group autonomy across diverse cultural contexts. In particular, the distinction between secular and religious school contexts in Israel may reflect differing cultural expectations regarding authority, obedience, and collective norms. These cultural factors may shape how Group Autonomy is constructed and experienced, and should therefore be considered when interpreting the findings and assessing their generalizability.

## 6. Conclusions

Taken together, the present findings suggest that the latency stage involves a developmental reorganization in which autonomy becomes increasingly situated within the peer group. By articulating Group Autonomy as a distinct developmental process, the study offers a reframing of middle childhood not merely as a continuation of individual independence, but as a period in which the group itself becomes a central arena of autonomous growth. This perspective extends models of autonomy development by drawing attention to a dimension that has received comparatively limited theoretical and empirical focus. Further research may clarify the conditions under which Group Autonomy can be supported in ways that promote both social cohesion and individual development.

## Data Availability

The data presented in this study are available on request from the corresponding author. The data are not publicly available due to privacy and ethical reasons.
